# R&D during public health emergencies: the value(s) of trust, governance and collaboration

**DOI:** 10.1136/bmjgh-2021-007873

**Published:** 2022-03-28

**Authors:** Rachel Alberta Katz, Fabio Salamanca-Buentello, Diego S Silva, Ross EG Upshur, Maxwell J Smith

**Affiliations:** 1 Faculty of Arts and Science, Institute for the History and Philosophy of Science and Technology, University of Toronto, Toronto, Ontario, Canada; 2 Faculty of Health Sciences, University of Western Ontario, London, Ontario, Canada; 3 Bridgepoint Collaboratory for Research and Innovation, Lunenfeld-Tanenbaum Research Institute, Toronto, Ontario, Canada; 4 Sydney School of Public Health, The University of Sydney, Camperdown, New South Wales, Australia; 5 Dalla Lana School of Public Health, University of Toronto, Toronto, Ontario, Canada

**Keywords:** COVID-19, Health policy, Health education and promotion, Control strategies

## Abstract

In January 2021, Dr Tedros Adhanom Ghebreyesus, director–general of the WHO, warned that the world was ‘on the brink of a catastrophic moral failure [that] will be paid with lives and livelihoods in the world’s poorest countries’. We are now past the brink. Many high-income countries have vaccinated their populations (which, in some cases, includes third and even fourth doses) and are loosening public health and social measures, while low-income and middle-income countries are struggling to secure enough supply of vaccines to administer first doses. While injustices abound in the deployment and allocation of COVID-19 vaccines, therapies and diagnostics, an area that has hitherto received inadequate ethical scrutiny concerns the upstream structures and mechanisms that govern and facilitate the research and development (R&D) associated with these novel therapies, vaccines and diagnostics. Much can be learnt by looking to past experiences with the rapid deployment of R&D in the context of public health emergencies. Yet, much of the ‘learning’ from past epidemics and outbreaks has largely focused on technical or technological innovations and overlooked the essential role of important normative developments; namely, the importance of fostering multiple levels of trust, strong and fair governance, and broad research collaborations. In this paper, we argue that normative lessons pertaining to the conduct of R&D during the 2014–2016 Ebola epidemic in West Africa provide important insights for how R&D ought to proceed to combat the current COVID-19 pandemic and future infectious disease threats.

**Summary box**

- The global response to the COVID-19 pandemic is characterised by global vaccine inequity. While the grossly inequitable global allocation of vaccines and other interventions like therapies and diagnostics deserves scrutiny, so too do the upstream structures and mechanisms of R&D leading to the development of such interventions.
- We can improve the R&D response to COVID-19 and future infectious disease threats by learning from past experiences. Yet, much of the ‘learning’ from past epidemics and outbreaks has largely focused on *technical* innovations, overlooking the essential role of *normative* innovations, namely, the importance of fostering multiple levels of trust, building strong and fair governance, and cultivating broad research collaborations.
- Cultivating these normative innovations to R&D from past epidemics and outbreaks, including the 2014–2016 Ebola epidemic in West Africa, is likely to play a key role in building trust in therapeutics, vaccines and diagnostics for COVID-19, particularly if (or when) high-income countries turn their attention from their domestic needs to supporting R&D efforts in low-income and middle-income countries.

## Introduction

In January 2021, Dr Tedros Adhanom Ghebreyesus, director–general of the WHO, began his address to WHO’s Executive Board with the bold statement that the world was ‘on the brink of a catastrophic moral failure [that] will be paid with lives and livelihoods in the world’s poorest countries’.^1^ We are now past the brink. Many high-income countries (HICs) have vaccinated their populations (which, in some cases, includes third and even fourth doses) and are loosening public health and social measures, while low-income and middle-income countries (LMICs) are struggling to secure enough supply of vaccines to administer first doses. While injustices abound in the deployment and allocation of COVID-19 vaccines, therapies and diagnostics, an area that has hitherto received comparatively inadequate ethical scrutiny concerns the upstream structures and mechanisms that govern and facilitate the research and development (R&D) leading to the development of such therapies, vaccines and diagnostics.

R&D spans many diverse activities. The focus taken in our paper draws on the strategy outlined in WHO’s R&D Blueprint for COVID-19 and the R&D activities addressed therein, which encompass a diverse but coordinated set of activities aiming to accelerate R&D for vaccines, therapies and diagnostics, undertaken by stakeholders such as scientists, research institutions, manufacturers, governments and regulatory bodies.^2 3^ These include traditional R&D activities like preclinical research, clinical research and manufacturing, but also activities like global research platforms, research priority setting, community engagement, data sharing, funding, and associated regulatory and ethical pathways.^4^ In addition, given our focus on the *ethics* of R&D, we also consider the broader social and political contexts within which R&D occurs, which includes the impacts that R&D activities may have for those contexts.

Many of the pressing R&D challenges faced during the COVID-19 pandemic are not new. Some were present during the 2014–2016 Ebola virus disease outbreak in West Africa.^4–6^ Controlling the Ebola epidemic required novel approaches to R&D—largely with respect to the speed and degree of communication required—to rapidly study and produce novel therapeutics and prophylactics to complement the public health measures deployed to curb the spread of disease.^4 7^ From collaboration between countries to efforts to encourage trust in local and international leaders, many innovations in the role of human relationships in R&D were key in curbing the Ebola epidemic.^8 9^

The R&D response during the West African Ebola epidemic demonstrated the speed with which therapeutics, vaccines, diagnostics and related R&D architecture can be developed to address outbreaks. Much can be learnt from these experiences and others for the world’s response to COVID-19 and future public health emergencies. Indeed, those evaluating the global response to the Ebola epidemic have subsequently urged for financial investments to jumpstart research innovations, facilitate manufacturing capacity and enhance information systems.^9^ However, as these examples illustrate, much of the ‘lessons learnt’ and associated recommendations have largely focused on *technical* or *technological* innovations informed by the West African Ebola epidemic and overlooked the essential role of *normative* (eg, ethical, relating to a value judgement) developments pertaining to R&D during public health emergencies,^5 6^ namely, the importance of fostering multiple levels of trust, building strong and fair governance, and cultivating broad research collaborations. We argue that these normative lessons provide important insights for how R&D ought to proceed to combat the current COVID-19 pandemic and future infectious disease threats.

Given the relevance of social and political contexts for the normative evaluation of R&D conducted during public health emergencies, we begin by briefly addressing key elements of the social and political context for the response to the West African Ebola epidemic. We then highlight the normative relevance of trust, governance and collaboration in the R&D response to that epidemic. Finally, we discuss how successes and setbacks in R&D related to the West African Ebola epidemic should inform R&D responses to the COVID-19 pandemic and future infectious disease threats.

## Social and political context of the West African Ebola virus disease epidemic

The successes and setbacks in R&D during the Ebola epidemic must be situated within the social and political contexts of Liberia, Guinea and Sierra Leone to understand how they relate to the central themes of trust, governance and collaborative partnerships explored in this paper. First, as others have noted, many of the interactions and instances of initial hostility towards international healthcare workers and foreign aid experienced in some cases were due to a legacy of colonialism in West Africa.^2^ For instance, in each of the countries that were primarily affected by the Ebola epidemic, aid and research (which are often difficult to disentangle) were largely directed through or governed by national institutions with direct ties to former colonial powers: France intervened in Guinea, the UK in Sierra Leone and American organisations in Liberia.^2^ These colonial legacies shaped how aid was initially offered and distributed in West Africa, as well as how research was designed and implemented.^10^ Underlying historical distrust of Western involvement led in some cases to local communities hesitating or refusing to comply with directions, thereby aiding the spread of the disease.^2^ Initial Ebola response strategies—including those for designing and implementing R&D—were not readily accepted by communities across the three countries and were erroneously framed as ‘resistance’ by media in HICs.^3^ While some Ebola-related initiatives were able to overcome this entrenched distrust, acknowledging the historical, colonial injustices visited on many LMICs by HICs is an important aspect of creating a strategy for R&D in response to a global public health emergency. This is particularly critical to keep in mind as much of the Global North vaccinates their populations while planning to ‘aid’ the Global South once the pandemic is ‘over’ in their home countries, and while new and ongoing clinical trials for novel COVID-19 vaccines and therapies are conducted in LMIC settings. In the following sections of this paper, we elaborate on why trust, governance and collaboration played vital roles in R&D during the West African Ebola epidemic, and how these normative developments for the conduct of R&D are crucial to COVID-19 and other disease threats.

## Trust

As a result of the colonial legacy in West Africa, fostering and building trust was difficult but paramount to the success of international involvement in R&D during the Ebola epidemic. While Guinea, Liberia and Sierra Leone each responded to the epidemic differently, trust—and a lack thereof—played a significant role in each case.

Trust was inhibited or otherwise eroded, for example, by approaches to data collection, storage and use during the West African Ebola epidemic.^11^ The generation and sharing of data are crucial for R&D, but their use—and by whom—is complicated and imbued by ethical considerations, and so must be carefully navigated for data generation and sharing to be successful.^12 13^ The Ebola epidemic created an avenue for data exploitation and hoarding, given the extensive exportation of biological samples and data from West Africa to Europe and North America.^4^ To date, these remain largely inaccessible to researchers and governments of Liberia, Guinea and Sierra Leone.^5^ Responsibility in the collection, storage, use and sharing of data was a key determinant of the successes or failures of the Ebola emergency response and related R&D.^14^ In order to learn from—rather than repeat—the poor data sharing examples observed during the Ebola epidemic, policies for sharing high-quality data that preserve and promote trust must be defended to enhance the quality and integrity of global COVID-19 R&D.^6^

For instance, leaders in Sierra Leone expressed confusion over healthcare workers’ need to take a blood sample from a woman who was dying from Ebola.^7^ The team that collected the sample was not treating the woman, nor was the village leader who expressed confusion made aware of why the blood sample was needed to confirm that the woman had Ebola.^7^ The already low level of trust was exacerbated by the spare-no-expense approach to controlling the Ebola outbreak, which was in stark contrast to the hands-off approach the international community generally employs for other diseases endemic to Africa.^7^ People living in communities affected by Ebola distrusted the large number of R&D initiatives implemented to control the Ebola epidemic, while no such action had been taken for other diseases, and some wondered if interventions such as taking blood from Ebola patients was part of a conspiracy to sell blood to international buyers.^7^ Families were hesitant to report cases of Ebola in their households because they distrusted the healthcare system, available interventions and ongoing R&D projects.^7^

To foster and build trust, the WHO has proposed global norms for public health emergencies that should be incorporated into R&D strategies for COVID-19 and future infectious disease threats, namely, timely and transparent sharing of data and results during public health emergencies as a global norm; timely publications of public disclosure information of relevance to public health emergencies; demonstrated responsibility by researchers for accuracy of shared data; data sharing as a default practice; and incentivising data sharing and enhancing data management and analysis expertise.^8^ Each of these strategies can promote trust, which can facilitate more successful R&D initiatives, largely because trust promotes collaboration, an important factor discussed later. Trust is a reciprocal process; two or more parties must engage in good faith in order to forge a trustworthy relationship that can further these aims in the context of R&D. The strategies outlined by the WHO ought to be considered and employed by all stakeholders involved in COVID-19 R&D and particularly those working with LMICs. These stakeholders include (but are not limited to) researchers in both HICs and LMICs, multilateral organisations, non-governmental organisations (NGOs) and communities at large.

## Good governance

Strong governance of R&D, especially during a public health emergency, often consists of the formation, coordination, and implementation of policies, guidelines and arrangements for participation, access to information and decision-making for the various R&D stakeholders operating within a given context.^15^ Two instruments of normative governance were especially important during the Ebola epidemic: regulations and rapid ethics review. This was in addition to the involvement of relevant international bodies with normative functions, such as the WHO (though, following the conclusion of the Ebola epidemic in 2016, the WHO was criticised for being ill-prepared to effectively lead the response to an epidemic or pandemic).^9^ A number of panels subsequently published reports critical of the WHO’s handling of the Ebola epidemic, calling for widespread reforms^16^; however, the WHO’s own advisory group on the Organisation’s emergency reform did not endorse some of these major changes.^16^ Other international governments were criticised for their interventions having more to do with protecting international interests than helping those who were actually sick at the time.^17^ This phenomenon has been referred to as the ‘pharmaceuticalisation’ of global health governance strategies; in the context of Ebola, this was critiqued as the interventions being approved at the time were seen as unlikely to be useful in curbing the epidemic.^17^ This highlights how instruments of normative governance (eg, research oversight and ethics review) ought to be guided by a principle of subsidiarity, which is itself predicated on efforts to build local capacity.^18^ As others have noted, this requires that research teams actively engage with affected communities while planning research to determine suitable trial designs that best reflect normative requirements.^11^

In the context of the outbreak, the governance of ethics review involved input and oversight from a number of different organisations, including the WHO’s advisory committee on ethics, along with local organisations on the ground in affected countries. Ultimately, many of the successes and failures in the response to the West African Ebola epidemic were a result of speed (or a lack thereof). Initially, trust levels were low in affected communities, which made it difficult to implement quarantine measures.^7^ Once that trust was further developed, it became easier for local governance measures to be implemented, such as the introduction of community leaders, who had more personal rapport with people living in small towns away from centralised governments.^2^ A similar situation unfolded on the ethics approval side of governance. Groups such as the Médecins sans Frontières (MSF) Ethics Review Board committed themselves to rapid project reviews even for complicated interventions, illustrating that rapid ethics response is possible (though as noted by Schopper and colleagues, future emergency ethics reviews must be completed faster than those completed during the Ebola epidemic).^16 19^

## Collaborative partnerships

Collaboration was a third normatively crucial factor in both international and more local or regional settings during the Ebola epidemic. As stated previously, different HICs were directly engaged with countries with whom they have a colonial history: France intervened in Guinea; the UK in Sierra Leone; and American organisations provided initial aid in Liberia.^2^ However, such interactions between HICs and LMICs were not always ‘true’ collaborations as they tended to prioritise the interests of HICs rather than those of the affected countries.^11^ The interactions embodied colonial legacies in which the balance of power tilted toward HICs and in which industrialised nations dictated the nature of engagement.

Several HICs framed their response in the context of their domestic agendas and prioritised effort in securing their national borders ahead of sending healthcare workers to West Africa with their engagements underpinned by selective historical alliances.^11^ Collaborative R&D partnerships were therefore being established or cultivated in a context were HICs were in some instances working for their own good. For instance, Nohrstedt and Baekkeskov identified five main political motivations that shaped HICs’ decisions to deploy healthcare workers in Liberia, Guinea and Sierra Leone, including threats to a foreign country’s own national security due to epidemics abroad, interdependence on medical and other resources, the presence/activity of international organisations and networks, domestic priority setting, and the influence of national institutions in intervening countries.^10^

This pernicious pattern of HIC-LMIC interaction is unfortunately familiar, but not every collaboration of this sort was forged to protect the interests of HICs. Notable among these was a unique data-related collaboration between Sierra Leone and the US Centres for Disease Control and Prevention (CDC). The Sierra Leone Ministry of Health — the body that owned the data collected in the country — partnered with the CDC primarily to consolidate Ebola data in order to share the locations of loved ones’ graves with surviving family members.^11 20^ This counterexample illustrates an instance where the decision to trust other groups, form good governance practices, and collaborate effectively led to a positive experience with data sharing.

## Lessons for COVID-19 and future public health emergencies

Experiences with past outbreaks, like the 2014–2016 Ebola epidemic, have led to significant technical innovations to the way in which we approach R&D for vaccines, therapeutics, and diagnostics in the context of public health emergencies. It is critical that we also learn normative lessons from our experiences with R&D during past public health emergencies. While scientific advances contribute valuable lessons to how we can better combat future public health emergencies, normative lessons show us how to better manage the human elements that are intrinsic to emergency management, and which facilitate or otherwise pave the way for the success of technical innovations. These lessons can inform individuals, groups, organisations and countries about how to best act in the face of a crisis and how to interact with other stakeholders. Failure to consider these normative aspects of R&D can lead to a failure to enact lasting change, both within the R&D space and in communities affected by health emergencies. Decision-makers in HICs must heed the many lessons learnt during the West African Ebola epidemic as efforts to vaccinate the global population against COVID-19 succeeds in HICs but flounders in LMICs. Studying the successes of certain relationships related to R&D during the Ebola epidemic and the conditions that led to their success is important, particularly where the inequities surrounding current vaccination efforts are concerned. Table 1 summarises these comparisons.

**Table 1:** Summary of the ways trust, governance and accountability apply to both the West African Ebola epidemic and the COVID-19 pandemic

	Trust	Governance	Collaboration
Importance to R&D	Trust enables the formation of strong governance measures, collaborative partnerships and ‘buy-in’ from local communities.	Changes to extant R&D governance during a health emergency enable the quicker processing of ethics review and aids in accelerating the development of R&D initiatives.	Collaboration is required in order to conduct research that equitably engages with the affected communities.
Positive effect on R&D during or following the West African Ebola epidemic	More timely and open data sharing was suggested as a way to build trust between researchers and communities.	Policies were implemented to streamline ethics review for interventions relevant to R&D that was beneficial to curbing the Ebola epidemic.	Collaborative efforts between Sierra Leone and the USA facilitated the dissemination of data on deceased loved ones to surviving family members.
Applicability to COVID-19 R&D	Researchers should engage with local leadership in order to build trust with affected communities, especially as COVID-19 is brought under control in HICs while the pandemic continues to rage in LMICs.	As HICs rein in their domestic COVID-19 case numbers, it is vital that international governments recognise that while the pandemic may be under control in HICs, the securitisation of their interests is insufficient to curb the pandemic.	In order to amend and improve on the lack of collaboration between HICs and LMICs early in the pandemic, researchers must initiate collaborations that actively engage individuals who have local expertise regarding their own communities’ needs.

HIC, high-income country; LMICs, low-income and middle-income countries; R&D, research and development.

Considering the challenges faced during the West African Ebola epidemic response, the global approach to curbing the COVID-19 pandemic must involve the development of trust on micro, meso and macro levels. This would involve actors including, but not be limited to, local and national politicians, organisations working in LMICs on COVID-19 R&D, and healthcare workers whose work may bridge both patient care and R&D projects. The development of a framework for R&D that addresses the importance of community engagement and transparency will play a key role in building trust in therapeutics, vaccines and diagnostics for COVID-19, particularly if (or when) HICs turn their attention to supporting R&D efforts in LMICs. This entails the involvement of local organisations and leadership by engaging health-related volunteer groups, such as those present in Sierra Leone during the Ebola epidemic.^13^ Volunteers in the healthcare sector liaised between healthcare providers and the general public, helped set up clinic and testing sites, and dispelled myths the public held about healthcare providers.^12 21^ Local and national groups have contextual knowledge about their communities’ health that must be acknowledged, respected and funded. Large international foundations have come under scrutiny for their decisions regarding donations and overall involvement in foreign aid during epidemics such as Ebola.^22^ During the Ebola epidemic, there was no clear or robust framework for ensuring accountability of independent agencies and NGOs such as the Bill and Melinda Gates Foundation (BMGF) and MSF for Ebola R&D initiatives.^23 24^ An accountability framework for R&D during global health emergencies is thus crucial to ensure all major stakeholders can be held to account and that more equitable outcomes from R&D are produced. Upholding a global health system accountability strategy is crucial for the success of COVID-19 R&D initiatives. The implication for R&D during the COVID-19 pandemic is that large organisations ought to consider allotting their financial contributions to local-level and national-level groups already working on the ground in communities, whether in LMICs or HICs, as opposed to establishing parallel R&D initiatives that may end up competing for the limited local health resources. As was observed during the Ebola epidemic, organisations such as the BMGF sought to fund the distribution of supplies, namely, through donations to United Nations agencies, along with ‘private and public sector partners to accelerate the development of therapies, vaccines, and diagnostics’.^25^ Global philanthropic organisations should avoid allocating resources to large international groups and NGOs and instead channel them to domestic groups and institutions that have already built strong bonds with local communities in LMICs hard hit by the COVID-19 pandemic.

A global pandemic naturally requires a global response. The WHO’s Access to COVID-19 Tools Accelerator (ACT-A) initiative has emerged as an international initiative that has significant potential to bring crucial vaccines, therapeutics and diagnostics to LMICs.^26^ While there are reasons to be hopeful that agreements via the ACT-A give LMICs a seat at global health policy tables, there is still reason for concern. Inclusion does not necessarily entail meaningful involvement, and it is possible that LMICs who have signed on to ACT Accelerator mechanisms, like COVAX, may hold little power as compared with the HICs in the development and implementation of policies surrounding the development of COVID-19 vaccines, diagnostics and therapeutics.^27^ The goal of COVAX, for instance, is to ensure equitable access to vaccines globally, so that self-financed and funded countries can access safe and effective vaccines.^21^ However, this egalitarian, collaborative approach to the distribution of COVID-19 vaccines can be compromised by funding shortages or offers for additional support of COVAX at an additional cost for the programme.^28^ This is pertinent as there is precedent in global health collaboration where LMICs have been largely included without being equally involved. For instance, global health initiatives (GHIs) in Africa were introduced to align and harmonise health interventions by governments and development partners.^29^ Since their introduction, however, GHIs have largely operated independently of the governments and bypassed country systems.^14^ Most importantly, GHIs often do not align with national strategic plans, and their specific earmarked funding has been used to impose restrictions on countries’ health development priorities.^14^ In the aftermath of the 2014–2016 Ebola epidemic, international efforts were made to strengthen global outbreak response systems, leading to the establishment of at least nine agencies, including Africa CDC and the Coalition for Emergency Preparedness Innovations.^16 30^ However, these initiatives were laden with significant disparities in the level of ownership granted to HICs as compared with those of LMICs; only three of nine initiatives reviewed significantly involved LMICs, while the others were largely sponsored and controlled by HICs.^16^

## Conclusions

Key normative lessons of the importance of fostering multiple levels of trust, building strong and fair governance, and cultivating broad research collaborations gleaned from R&D efforts during the West African Ebola epidemic should inform the R&D response to the COVID-19 pandemic, with particular emphasis on mitigating the growing disparities and inequities between HICs and LMICs. It is essential to build trust with local communities and researchers in affected countries. Legitimate collaborations between HICs and LMICs should emphasise justice and equity and should prioritise the needs of populations in LMICs. Crucially, it should be clear that local communities in LMICs have expertise and extant relationships that should be acknowledged, respected and included in R&D efforts related to the pandemic. Efforts to operationalise these normative lessons for R&D ought to be guided by a principle of subsidiarity, which is predicated on efforts to build local capacity for research collaboration and governance. The issues examined in this paper can help build the foundation for more efficient and equitable R&D approaches to the public health emergencies that will inevitably surface in the future.

## Data Availability

There are no data in this work.

## References

[1] WHO Director-General’s opening remarks at 148th session of the Executive Board [Internet], 2021. Available: https://www.who.int/director-general/speeches/detail/who-director-general-s-opening-remarks-at-148th-session-of-the-executive-board

[2] About R&D Blueprint [Internet], 2022. Available: https://www.who.int/teams/health-product-and-policy-standards/about/blueprint

[3] A Coordinated Global Research Roadmap [Internet], 2022. Available: https://www.who.int/publications/m/item/a-coordinated-global-research-roadmap

[4] World Health Organization. An R&D Blueprint for Action to Prevent Epidemics: Accelerating R&D and Saving Lives [Internet], 2017. Available: https://cdn.who.int/media/docs/default-source/blue-print/an-randd-blueprint-for-action-to-prevent-epidemics-update-2017.pdf

[5] Smith MJ, Upshur REG. Learning lessons from COVID-19 requires recognizing moral failures. J Bioeth Inq 2020;17:563–6. 10.1007/s11673-020-10019-6 32840847 PMC7445711

[6] Smith MJ, Upshur REG. Ebola and learning lessons from moral failures: who cares about ethics? Public Health Ethics 2015;8:phv028–18. 10.1093/phe/phv028 PMC7107108 32288786

[7] World Health Organization. Report of the Ebola Interim Assessment Panel [Internet], 2015. Available: https://www.who.int/publications/m/item/report-of-the-ebola-interim-assessment-panel-july-2015

[8] United Nations Development Program. Getting beyond zero - Early recovery and Resilience Support Framework: Guinea, Liberia and Sierra Leone | United Nations Development Programme [Internet], 2022. Available: https://www.undp.org/publications/getting-beyond-zero-early-recovery-and-resilience-support-framework-guinea-liberia-and

[9] Gostin LO, Tomori O, Wibulpolprasert S, et al. Toward a common secure future: four global commissions in the wake of Ebola. PLoS Med 2016;13:e1002042. 10.1371/journal.pmed.1002042 27195954 PMC4873000

[10] Nohrstedt D, Baekkeskov E. Political drivers of epidemic response: foreign healthcare workers and the 2014 Ebola outbreak. Disasters 2018;42:41–61. 10.1111/disa.12238 28440550

[11] Presidential Commission for the Study of Bioethical Issues. Ethics and Ebola: Public Health Planning and Response [Internet]. Washington D.C, 2015. Available: https://bioethicsarchive.georgetown.edu/pcsbi/sites/default/files/Ethics-and-Ebola_PCSBI_508.pdf

[12] National Academies of Sciences, Engineering, and Medicine. Data Matters: Ethics, Data, and International Research Collaboration in a Changing World: Proceedings of a Workshop [Internet]. Sloan SS, Alper J, editors. Washington, DC: The National Academies Press, 2018. Available: https://www.nap.edu/catalog/25214/data-matters-ethics-data-and-international-research-collaboration-in-a 30601606

[13] Lencucha R, Bandara S. Trust, risk, and the challenge of information sharing during a health emergency | Globalization and Health | Full Text [Internet], 2021. Available: https://globalizationandhealth.biomedcentral.com/articles/10.1186/s12992-021-00673-9 10.1186/s12992-021-00673-9 PMC7890381 33602281

[14] Modjarrad K, Moorthy VS, Millett P, et al. Developing global norms for sharing data and results during public health emergencies. PLoS Med 2016;13:e1001935. 10.1371/journal.pmed.1001935 26731342 PMC4701443

[15] Eccleston-Turner M, Upshur R. Inter-Institutional relationships in global health: regulating coordination and ensuring accountability. Global Health Governance 2018;12:83–99.

[16] Mackey TK. The Ebola Outbreak: Catalyzing a "Shift" in Global Health Governance? BMC Infect Dis 2016;16:699. 10.1186/s12879-016-2016-y 27881085 PMC5121963

[17] Marcis FL, Enria L, Abramowitz S. Three acts of resistance during the 2014–16 West Africa Ebola epidemic: a focus on community engagement. J Humanit Aff 2019;1:23–31.

[18] Brakman S-V. Guiding principles of community engagement and global health research: solidarity and Subsidiarity. Am J Bioeth 2020;20:62–4. 10.1080/15265161.2020.1745946 32364486

[19] Schopper D, Ravinetto R, Schwartz L, et al. Research ethics governance in times of Ebola. Public Health Ethics 2017;10:49–61. 10.1093/phe/phw039 28567113 PMC5444563

[20] Gorina Y, Redd JT, Hersey S, et al. Ensuring ethical data access: the Sierra Leone Ebola database (SLED) model. Ann Epidemiol 2020;46:1–4. 10.1016/j.annepidem.2020.04.001 32532366 PMC7443172

[21] McMahon SA, Ho LS, Scott K, et al. "We and the nurses are now working with one voice": How community leaders and health committee members describe their role in Sierra Leone's Ebola response. BMC Health Serv Res 2017;17:495. 10.1186/s12913-017-2414-x 28720090 PMC5516346

[22] Woolhouse MEJ, Rambaut A, Kellam P. Lessons from Ebola: improving infectious disease surveillance to inform outbreak management. Sci Transl Med 2015;7:307rv5. 10.1126/scitranslmed.aab0191 PMC5819730 26424572

[23] Freudenthal E. Ebola’s lost blood: row over samples flown out of Africa as “big pharma” set to cash in. The Telegraph [Internet], 2019. Available: https://www.telegraph.co.uk/global-health/science-and-disease/ebolas-lost-blood-row-samples-flown-africa-big-pharma-set-cash/

[24] Bill & Melinda Gates Foundation Commits $50 Million to Support Emergency Response to Ebola - Bill & Melinda Gates Foundation [Internet], 2021. Available: https://www.gatesfoundation.org/ideas/media-center/press-releases/2014/09/gates-foundation-commits-$50-million-to-support-emergency-response-to-ebola

[25] Afolabi MO, Folayan MO, Munung NS, et al. Lessons from the Ebola epidemics and their applications for COVID‐19 pandemic response in sub‐Saharan Africa. Developing World Bioethics. 10.1111/dewb.12275 PMC7404531 32654261

[26] Berkley S. COVAX explained [Internet], 2020. Available: https://www.gavi.org/vaccineswork/covax-explained

[27] Smith MJ, Ujewe S, Katz R, et al. Emergency use authorisation for COVID-19 vaccines: lessons from Ebola. Lancet 2020;396:1707–9. 10.1016/S0140-6736(20)32337-0 33160452 PMC7836825

[28] Richards P, Mokuwa E, Welmers P, et al. Trust, and distrust, of Ebola treatment centers: a case-study from Sierra Leone. PLoS One 2019;14:e0224511. 10.1371/journal.pone.0224511 31790420 PMC6886773

[29] Mwisongo A, Nabyonga-Orem J. Global health initiatives in Africa - governance, priorities, harmonisation and alignment. BMC Health Serv Res 2016;16 Suppl 4:212. 10.1186/s12913-016-1448-9 27454542 PMC4959383

[30] Ravi SJ, Snyder MR, Rivers C. Review of international efforts to strengthen the global outbreak response system since the 2014-16 West Africa Ebola epidemic. Health Policy Plan 2019;34:47–54. 10.1093/heapol/czy102 30624680 PMC7107495

